# Ketogenic diet influence on the elemental homeostasis of internal organs is gender dependent

**DOI:** 10.1038/s41598-023-45611-4

**Published:** 2023-10-27

**Authors:** Kamil Kawon, Marzena Rugiel, Zuzanna Setkowicz, Katarzyna Matusiak, Aldona Kubala-Kukus, Ilona Stabrawa, Karol Szary, Zuzanna Rauk, Joanna Chwiej

**Affiliations:** 1 Faculty of Physics and Applied Computer Science, AGH University of Krakow, Kraków, Poland; 2 Institute of Zoology and Biomedical Research, Jagiellonian University, Kraków, Poland; 3 Institute of Physics, Jan Kochanowski University, Kielce, Poland; 4 Holy Cross Cancer Center, Kielce, Poland

**Keywords:** Biophysics, Chemical biology, Biomarkers, Molecular medicine, Risk factors

## Abstract

The ketogenic diet (KD) is a low-carbohydrate and high-fat diet that gains increasing popularity in the treatment of numerous diseases, including epilepsy, brain cancers, type 2 diabetes and various metabolic syndromes. Although KD is effective in the treatment of mentioned medical conditions, it is unfortunately not without side effects. The most frequently occurring undesired outcomes of this diet are nutrient deficiencies, the formation of kidney stones, loss of bone mineral density, increased LDL (low-density lipoprotein) cholesterol levels and hormonal disturbances. Both the diet itself and the mentioned adverse effects can influence the elemental composition and homeostasis of internal organs. Therefore, the objective of this study was to determine the elemental abnormalities that appear in the liver, kidney, and spleen of rats subjected to long-term KD treatment. The investigation was conducted separately on males and females to determine if observed changes in the elemental composition of organs are gender-dependent. To measure the concentration of P, S, K, Ca, Fe, Cu, Zn and Se in the tissues the method of the total reflection X-ray fluorescence (TXRF) was utilized. The obtained results revealed numerous elemental abnormalities in the organs of animals fed a high-fat diet. Only some of them can be explained by the differences in the composition and intake of the ketogenic and standard diets. Furthermore, in many cases, the observed anomalies differed between male and female rats.

## Introduction

The ketogenic diet (KD) is characterized by the complete elimination or the reduction of carbohydrate intake, and the main source of energy during its use are fats and proteins^1^. During a normal diet, the glycolysis process dominates, and the precursor for the production of adenosine triphosphate (ATP) in the body is pyruvate, which participates in the Krebs cycle. In case of KD, however, the intensity of beta-oxidation of fatty acids in the liver and the synthesis of ketone bodies (acetoacetate, beta-hydroxybutyrate and acetone) significantly increases^2^. The metabolism of ketone bodies is similar to the metabolism of carbohydrates, with the difference that instead of pyruvate, the precursor for acetyl-CoA, which enter the Krebs cycle, is beta-hydroxybutyrate^2^.

Although KD has been used for many years as an individual or adjuvant therapy in the treatment of various diseases, the mechanisms of its curative effect are still not fully understood. For around hundred years KD has been utilized for the management of refractory seizures. It is mostly used to treat pediatric epilepsies but gives satisfactory results also in adolescents and adults^3–5^. The postulated mechanism of anticonvulsant effect of KD consists in the fact that as a low-carbohydrate diet it leads, by reducing the intensity of the glycolysis process, to a decrease in ATP available in the cytoplasm. The decreased level of ATP in the cytoplasm of nerve cells results in the opening of ATP-dependent potassium channels, which increases the membrane potential and causes a reduced response of cells to the stimuli^6^. In addition, during the use of KD, the concentration of gamma-aminobutyric acid (GABA), which is an inhibitory neurotransmitter in the central nervous system, increases^6^.

The preclinical studies based on animal models have also demonstrated anti-angiogenic, anti-invasive, and pro-apoptotic action of KD in mice with malignant brain cancer^7^. KD enhanced tumor-reactive immune response and sensitized tumors to standard of care therapies used for glioblastoma multiforme (GBM)^8^. Used as adjuvant treatment to radiotherapy it led to additional reduction of tumor growth and increased survival of animals with glioma^9^. Glioma cells are characterized by increased energy demand^10^. It is also postulated that, unlike normal glial cells and neurons, they are not able to use ketone bodies and survive the metabolic stress caused by glucose deficiency^10^. Therefore, KD changing the main source of energy for the metabolism from glucose to ketone bodies can negatively affect cancer cells, saving normal ones, and thus selectively inhibit tumor growth and improve the survival of patients suffering from GBM.

Despite the confirmed or expected therapeutic efficiency of KD in case of various diseases, this diet is not free from undesirable reactions and the most common side effects of KD include nausea, vomiting, headaches and dizziness, fatigue, insomnia, difficulties in tolerating exercise, and constipation. In turn long-term undesirable effects involve fatty liver, protein, vitamin and mineral deficiencies and the appearance of kidney stones^11^. Therefore, the aim of this study was to determine the influence of the use of KD on the elemental homeostasis of organs engaged in fat metabolism and/or those in which disorders associated with long-term exposure to ketone bodies during KD are observed. The investigation was carried out on rats. The liver, kidneys and spleen were selected for the study. In the liver, beta-oxidation of fatty acids occurs and ketone bodies are produced, and long-term use of KD can lead to fatty liver^12^. The kidneys are responsible for filtering the blood from harmful metabolites, and long-term ketosis can result in hypercalciuria, as a result of which kidney stones can accumulate in the organ^13^. Additionally, the effect of KD on the elemental composition of the spleen was studied. Because of significant gender differences in the range of the overall organism functioning and hormonal balance^14^ as well as the regulated by sex hormones lipid and glucose metabolism^15^, the study was done, separately, on males and females.

To examine the elemental abnormalities appearing in the digested samples of rat organs the total reflection X-ray fluorescence method (TXRF)^16^ was applied. The mentioned instrumental technique of elemental analysis is characterized by the possibility of simultaneous analysis of the content of many elements, small amount of material needed for analysis, short determination time and good detection limit at the ng/g level.

## Results

### Glucose/ketone bodies concentration in blood of ketogenic and standard diet fed animals

**Figure 1:**
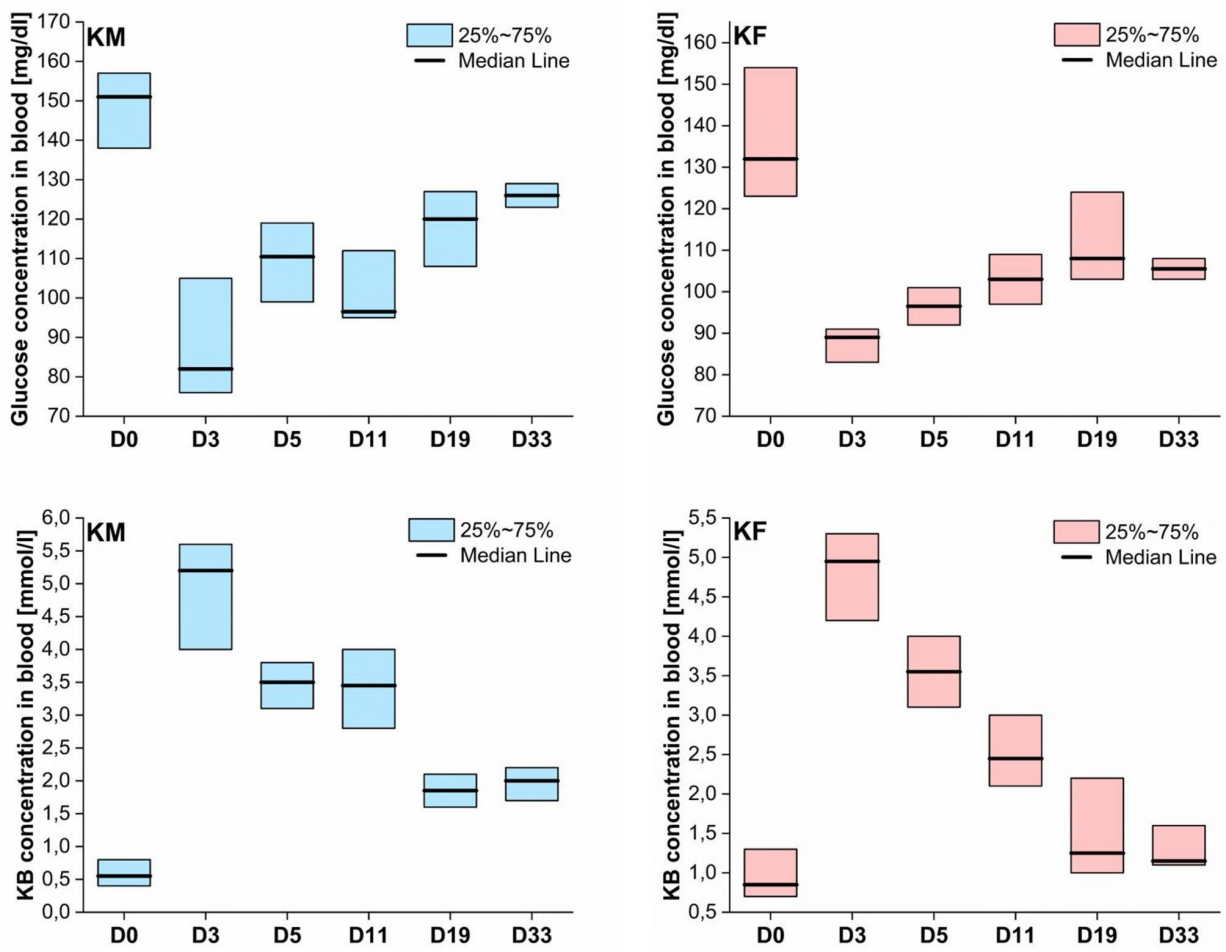
Blood concentration of glucose (upper row) and ketone bodies (lower row) in male (blue) and female rats fed with ketogenic fodder before (D0) and on the days: 3, 5, 11, 19 and 33 of the dietary treatment (D3, D5, D11, D19 and D33, respectively).

The median blood concentration of glucose in male rats fed with standard laboratory diet was $137 \pm 29\; {\text{mg/dl}}$, and in females $125 \pm 20\; {\text{mg/dl}}{.}$ In turn, the concentration of ketone bodies equaled to $0.51\; \pm \;0.33\; {\text{mmol/l}}$ and $0.72\; \pm \;0.43\; {\text{mmol/l}}$ for male and female controls, respectively. In case of KD fed animals the mentioned above blood parameters were controlled before an experiment (D0) and on the days 3, 5, 11, 19 and 33 of the dietary treatment (marked as D3, D5, D11, D19 and D33, respectively). The progress of the changes of the glucose and ketone bodies levels in blood of animals fed with high fat fodder was presented in the Fig. 1.

As shown in Fig. 1, after 3 days of KD use, a significant increase of the ketone bodies concentration and diminished glucose level in the blood of animals was observed. In the following days of the experiment, the level of ketone bodies slowly decreased, whilst that of glucose increased. Nevertheless, at the end of the experiment, animals fed a high-fat diet were still in a state of dietary ketosis, with the blood ketone bodies concentration higher than $1 \;{\text{mmol/l}}$^17^.

### Elemental composition of liver, kidney and spleen of ketogenic and standard diet fed animals

**Figure 2:**
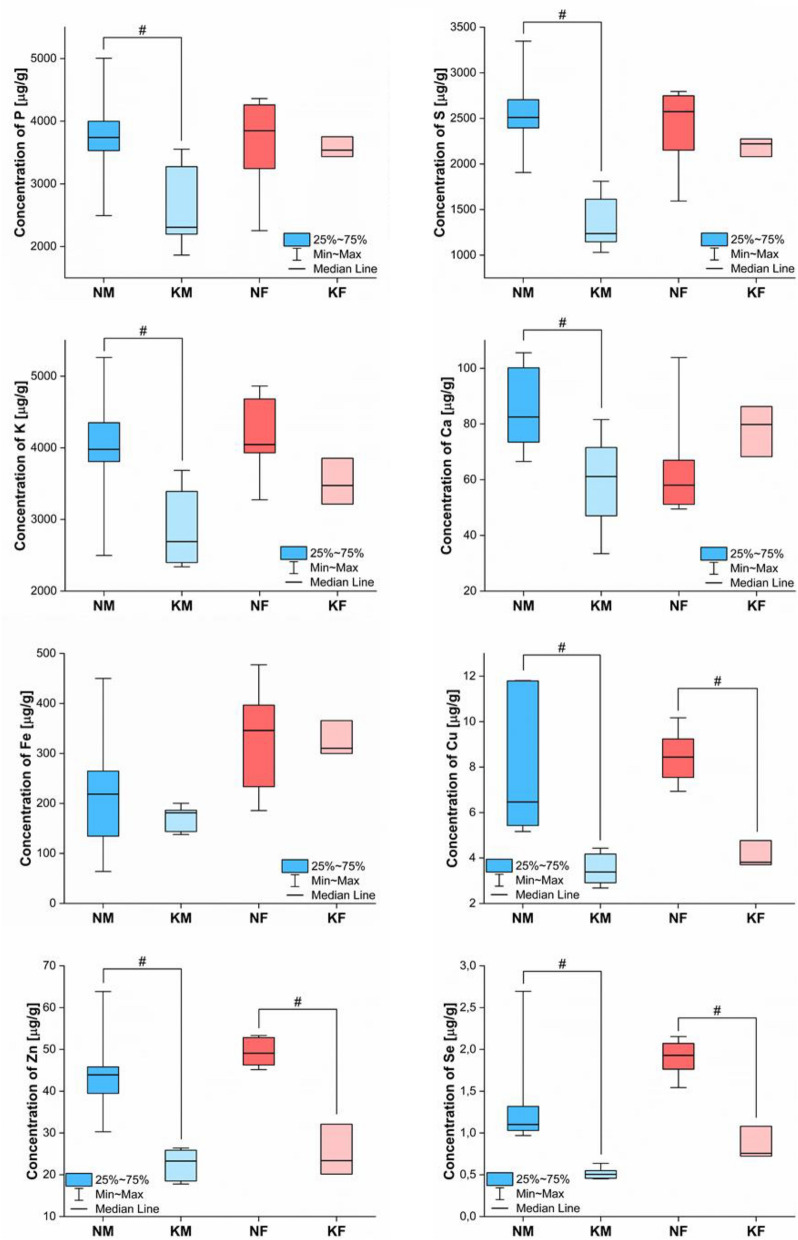
Box-and-whiskers plots presenting the ranges of element concentrations [μg/g] in livers taken from male (blue) and female (red) rats fed with ketogenic (groups KM and KF) and standard diets (groups NM and NF). Median (line), interquartile range (box) and minimal-maximal values (whiskers) are marked. The statistically significant differences determined with Mann–Whitney *U* test (*p*-value < 0.05) between KD treated animals and controls of given gender are signed with #.

**Figure 3:**
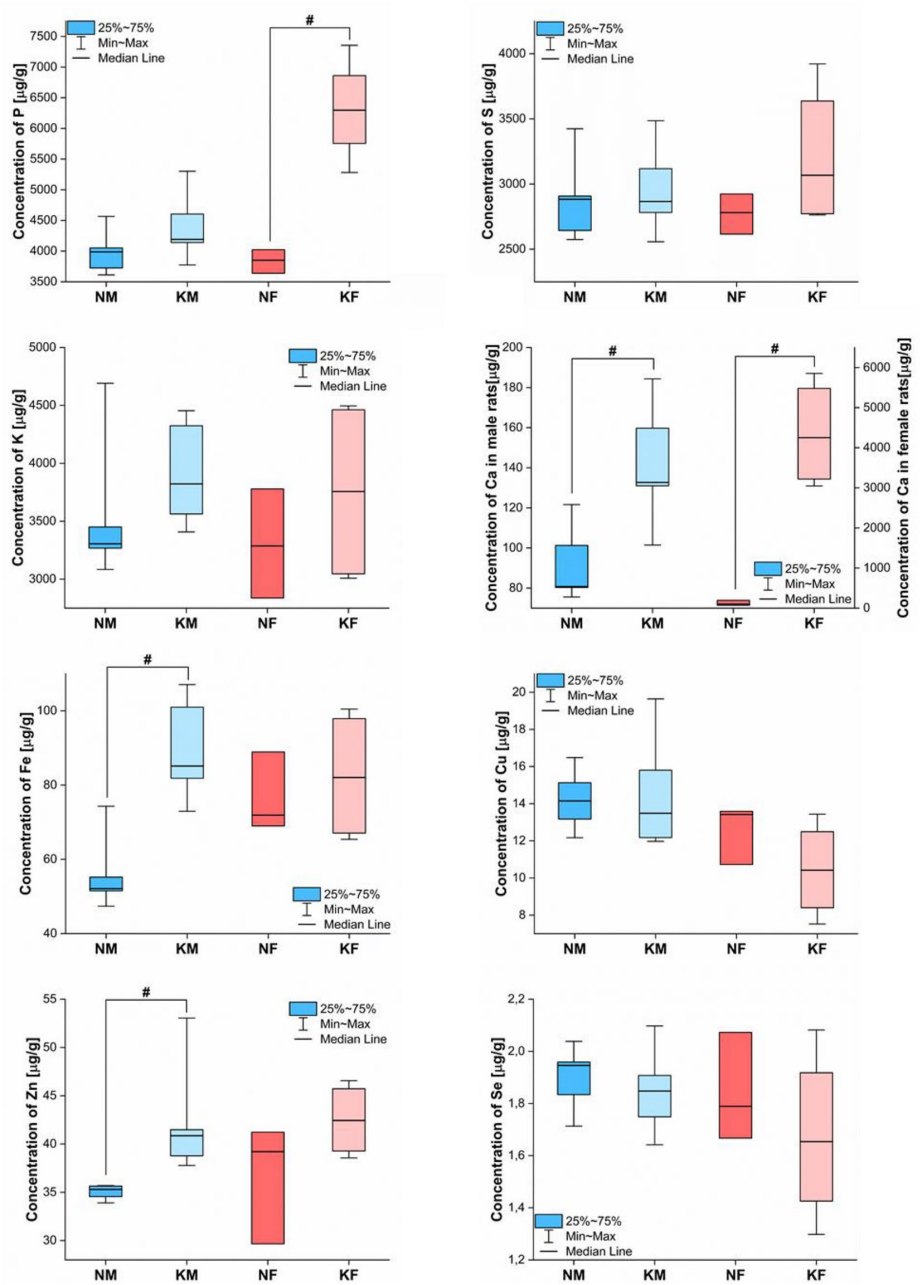
Box-and-whiskers plots presenting the ranges of element concentrations [μg/g] in kidneys taken from male (blue) and female (red) rats fed with ketogenic (groups KM and KF) and standard diets (groups NM and NF). The statistically significant differences determined with Mann–Whitney *U* test (*p*-value < 0.05) between KD treated animals and controls of given gender are signed with #.

**Figure 4:**
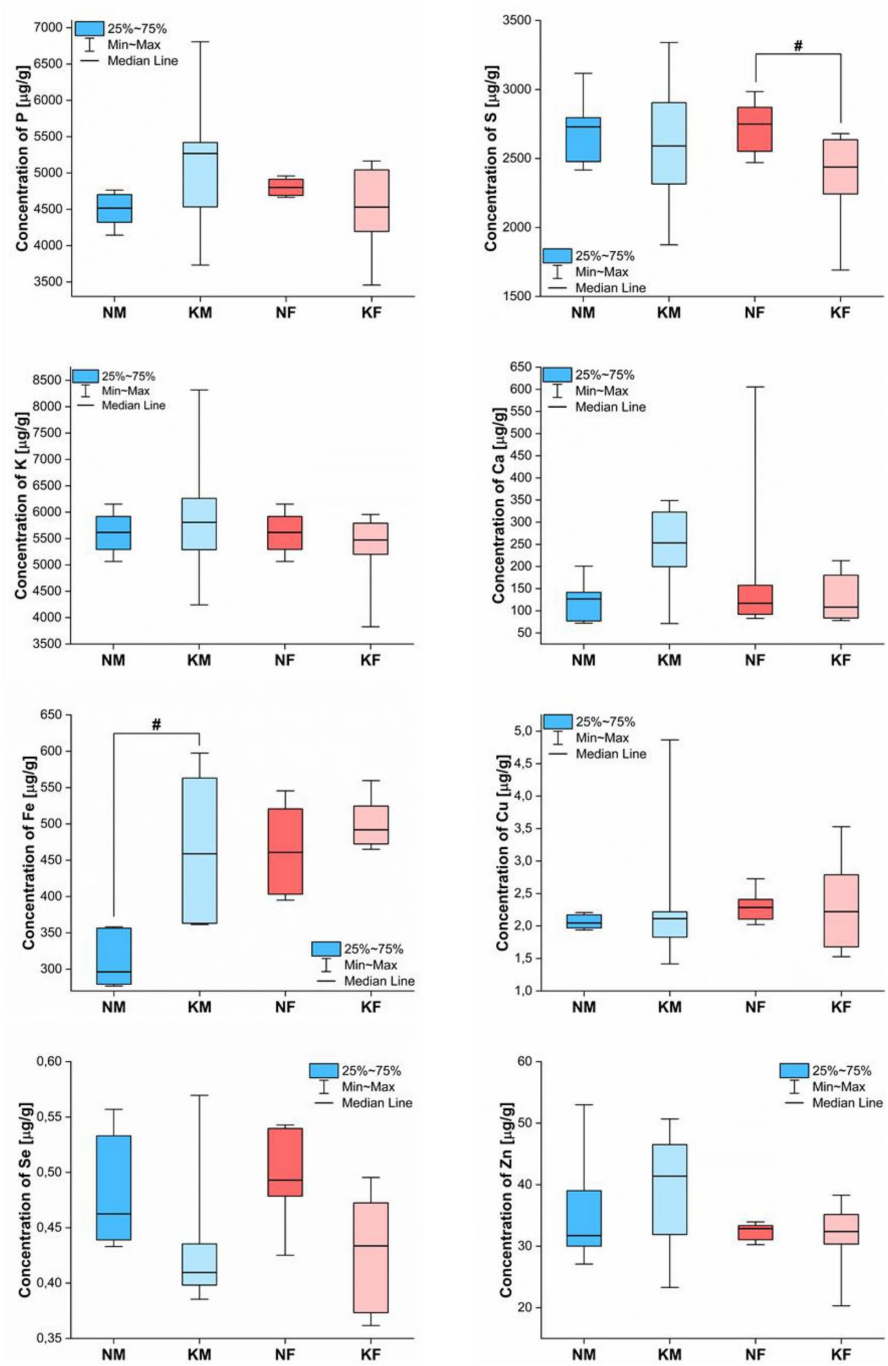
Box-and-whiskers plots presenting the ranges of element concentrations [μg/g] in spleens taken from male (blue) and female (red) rats fed with ketogenic (groups KM and KF) and standard diets (groups NM and NF). The statistically significant differences determined with Mann–Whitney U test (*p*-value < 0.05) between KD treated animals and controls of given gender are signed with #.

Using the TXRF method, the concentration of P, S, K, Ca, Fe, Cu, Zn and Se in the liver, kidney and spleen of each examined rat was determined. These data were used to prepare box-whisker plots (Figs. 2, 3 and 4) presenting the scatter of the results obtained for 4 tested animal populations, namely, for male and female rats on KD (marked as KM and KF groups, respectively) and for the animals of both sexes on the standard laboratory diet (marked as NM and NF groups). In addition, in the Figs. 2, 3 and 4, the results of the Mann–Whitney *U* test showing statistically significant differences between animals of a given gender on KD and an ordinary laboratory diet were placed. In turn, in Table S1 of Appendix, the detection limits (LOD) of the measured elements calculated for 3 studied organs were presented. As one can notice from this Table, the best LOD values were obtained for kidneys and they were the lowest for Se ($0.0373 \;{{\mu g/g}}$) and the highest for P ($12.87\; {{\mu g/g}}$).

As it can be seen in Fig. 2, KD significantly affects the elemental homeostasis of the liver. Apart from Fe, the level of all the analyzed elements is lower in the organ of KD-fed males than in those fed a standard diet. Interestingly, in case of female rats, a statistically significant decrease of the concentration in this organ was found only for Cu, Zn and Se. The reduced accumulation of some of the tested elements in the liver can be explained by their reduced supply in KD (the details in the Fig. S1 of Appendix) and lower fodder weight consumed by rats on KD compared to those on a standard diet. However, these are not the only sources of elemental abnormalities observed in the liver, which is indicated, for example, by gender differences in the accumulation of P, S, K and Ca in the organ.

Much greater gender dissimilarities were observed when analyzing the effect of KD on the elemental composition of the kidneys. Elevated concentrations of Fe, Zn and Ca were found in males, while in females the levels of P and Ca increased. Although renal Ca accumulation was higher after KD in both sexes, this effect was definitely stronger in female rats.

The least number of statistically significant differences in elemental composition, between animals on a ketogenic and standard diet, were recorded for spleen. After a high-fat diet, male and female rats showed, respectively, increased Fe and S level in the organ.

## Discussion and conclusions

The purpose of this paper was determination of the element abnormalities that appear in the rat internal organs as a result of the treatment with KD. We examined, also, how animal gender influences observed modifications of the element homeostasis. The liver, kidney and spleen were selected for the study, and for the elemental analysis of the digested tissues TXRF method was applied. With the use of it, the concentrations of P, S, K, Ca, Fe, Cu, Zn and Se were determined in the liquid organ samples.

The obtained results showed a large number of elemental abnormalities in the organs taken from animals fed with the high fat fodder. In some cases, the found anomalies were different for male and female rats. Analysis of liver samples showed lower concentration of all elements, except Fe, for male rats fed with the ketogenic fodder. In case of females, the effect was observed only for higher-Z elements, namely, for Cu, Zn and Se. Comparing the content of the examined elements in ketogenic and standard diet (Fig. S1 of the Appendix), one can see that the concentration of most of them (besides K and Zn) in the high fat diet is much lower than in the normal one. This factor, together with the fact that the mass of the fodder consumed by animals is also lower in case of high fat diet, could to some degree explain observed disorder of element homeostasis of the liver. However, different pattern of elemental abnormalities found between male and female rats, normal liver Fe level despite of diminished content of the element in ketogenic fodder, the lack of abnormalities in the range of low-Z elements for female rats, suggest that the diminished level of measured elements in the fodder is not the only reason of the found liver anomalies.

The observed lower concentration of P and Ca in the liver of male rats on KD may be connected with the decreased insulin level during the intake of poor in sugars chow^18^. The insulin influences the level of D vitamin, which plays an important role in the process of absorption of P and Ca from the intestines^19^. The disturbed absorption and content of these elements in blood may result in their diminished liver concentration. After the treatment of male rats with KD, the decreased K content in the liver was, also, found. Such a result may be an effect of diminished availability of glycogen^20^ in liver and muscles^21^. K is an important cofactor for glycogen phosphorylase, an enzyme participating in the glycogenolysis process^22^. Its reduced level may be, therefore, connected with the limited requirement for the enzyme when low levels of glycogen are observed^23^.

The diminished level of Cu, Zn and Se within the liver was found in animals of both genders that were fed with KD. For Cu and Se, the effect may be explained, among others, by the decreased content of these elements in the high fat diet and lower ketogenic fodder intake in comparison with the standard one. The lower levels of trace elements in serum of adults after the long-term treatment with KD were observed previously^24^ and the found effect depended on the used diet formula^25^.

KD used to treat refractory seizures in children was leading to the appearance of kidney stones^13,26^. Kidney stones are mostly formed from uric acid. However, there are also some that contain minerals such as struvite, apatite and brushite, as well as those that are made of calcium phosphate mono- or dihydrates^27,28^ and calcium salt of oxalic acid^29^. The elevated risk of kidney stones formation during KD, together with their typical elemental composition, seem to elucidate the observed increases of P and Ca concentration in kidneys of female rats. An explanation for the elevated Ca levels found in animals of both genders may be also the occurrence of hypercalciuria as a side effect of KD treatment^13^.

Male rats presented higher concentration of Zn in kidneys. Together with Cu, the mentioned element is a part of Cu,Zn-superoxide dismutase^30^ belonging to a family of enzymes that catalyze the dismutation of the superoxide radicals^31^. Increased level of the enzyme in kidneys of animals fed with KD might be a result of the inflammation processes occurring in the organ and connected with them reactive oxygen species release^32^. In such a case, increased kidney Zn accumulation should correlate with the elevated Cu concentration within the organ. Such correlation was, however, not found.

Watanabe et al.^33^ observed that the use of KD may result in the chronic kidney disease. In patients suffering from kidney diseases, renal tubules are exposed to a high concentration of Fe owing to increased glomerular filtration of iron and iron-containing proteins. What is more, the levels of intracellular iron may increase when glomerular and renal tubular cells are injured^34^. The mentioned phenomena connected with the chronic kidney disease may be, therefore, the source of increased concentration of Fe in kidneys of male rats fed with KD.

The elevated Fe concentration in the spleen of male rats might be a result of increased red blood cells production. However, according to Nazarewicz et al.^35^ the use of KD treatment does not have a positive effect on the blood parameters including red blood cells and hemoglobin level. The systemic iron-regulatory hormone is hepatic peptide hepcidin^36^. The hepcidin synthesis is transcriptionally regulated by extracellular and intracellular iron concentrations, and its increased concentration in plasma is observed in iron-restrictive anemias including those connected with inflammation, chronic kidney disease and some cancers^37^. Arsyad et al.^32^ showed that long-term treatment of rats with KD may lead to anemia. During anemia hepcidin, being the negative regulator of hemopoiesis^36^, blocks the release of Fe from spleen macrophages^37^.

Most of the literature data show that KD treatment globally diminishes the level of the oxidative stress in the body^38,39^. This does not exclude, however, the possibility of its local occurrence in the answer to, for example, the high-fat diet induced cellular damage and/or inflammation process, especially in liver^32,40^. Globally reduced level of the oxidative stress limits the need to produce glutathione by the spleen^41^. In turn, the diminished glutathione level, being the major reservoir of non-protein reduced sulfur, may be the source of the decreased level of this element observed in the spleen for female rats. Se is an element necessary for the proper functioning of glutathione peroxidase enzymes. Therefore, decreased accumulation of Se within spleen observed for animals fed with high fat diet, may also be result of the positive effect of KD on the level of oxidative stress^42^. However, it should be noted that the high-fat feed used in the experiment was characterized by a very low content of this element, what may also be the source of found abnormalities.

Summarizing, the treatment with KD may significantly influence the elemental homeostasis of internal organs. Only some of the observed elemental anomalies may be explained by differences in the composition and intake of the high fat fodder, and at least some of them seem to be gender dependent.

## Materials and methods

### Animals

All methods are reported in the paper in accordance with ARRIVE guidelines (https://arriveguidelines.org). The animals used in this study were Wistar rats originating from the husbandry of the Department of Experimental Neuropathology of the Institute of Zoology and Biomedical Research, Jagiellonian University in Krakow. All animal studies were conducted in accord with the international standards and under approval of the 2nd Local Institutional Animal Care and Use Committee in Krakow. They were done under agreement no. 316/2020. Twelve male and twelve female rats were included in the study. On the day 27th of postnatal development, animals of both genders were divided into 2 equal subgroups which from that time, for the next 33 days, were fed either with the ketogenic or standard laboratory fodder. Once a week, the rats were weighed. In KD fed animals the state of dietary ketosis was controlled and for this purpose the levels of ketone bodies and glucose in their blood were measured at the beginning of the experiment (D0) and on the chosen days of the dietary treatment (D3, D5, D11, D19 and D33).

On the day 60th of life, the rats received a lethal dose of pentobarbital and then were perfused transcardially with physiological saline solution of high analytical purity. The organs (liver, kidney and spleen) taken from animals were immediately frozen in liquid nitrogen and till the digestion procedure kept in the temperature not higher than – 20 °C.

### Ketogenic and standard laboratory diet

The ketogenic diet (EF R/M with 80% Fat—ketogenic), enriched in fat, was purchased from ssniff®, whilst normal laboratory diet (Labofeed H Standard) from Morawski company. For the ketogenic diet, 94% of metabolizable energy (ME) came from fats and the remaining 6% from proteins and carbohydrates. On the other hand, in case of the standard diet, 60% of ME was provided in the form of carbohydrates, 30% as proteins and the remaining 10% as fats. The animals had the access to food and water *ad libido* but the daily intake of the chow was controlled.

### Sample preparation

The element analysis using the TXRF method was performed for liquid organ samples. To obtain them, each collected organ, in a separate Teflon vessel (DAP100), was subjected to the microwave assisted acid digestion. The mineralization procedure was done in the 65% nitric acid of high purity (Suprapur, Merck) with the use of Speed Wave 4 system (Berghof). The typical volume of acid used for digestion was 2.5 ml per 1 g of tissue. The obtained liquid organ samples were poured into sterile eppendorf tubes and were kept under refrigeration conditions until the measurements were taken.

To estimate the elemental composition of ketogenic and standard diet with TXRF method, the liquid samples of the fodders were prepared. First, 100 g of each diet was initially homogenized, and then 6 samples with the mass of 200 mg were taken and subjected to microwave assisted digestion in 5 ml of the 65% nitric acid. For each of 6 samples of particular fodder, the mineralization procedure was done in a separate Teflon vessel.

### TXRF measurements

The quantitative elemental analysis was based on the internal standard method and gallium (Ga) was used for this purpose. 50 μl of 1000 mg/l Ga solution (Gallium ICP standard in HNO_3_ 2–3% 1000 mg/l Ga Certipur®, Merck) was added to the 1.5 ml of the tissue digest and mixed thoroughly. 6 μl of such solution was taken and spotted on the clean quartz glass carrier which was then dried on a heating plate. For each organ sample 3 independent replicates were made.

The measurements were carried out in the Laboratory of X-ray Methods of the Centre for Research and Analysis at the Jan Kochanowski University. S2 PICOFOX TXRF spectrometer (Bruker Nano) equipped with the Mo-anode X-ray tube was used for the study. The tube voltage was 50 kV, whilst its current 0.6 mA. The acquisition time of spectrum was 1000 s. The obtained spectral data were analysed using Picofox Spectra 7 (Bruker Nano) software.

### Quantitative element analysis

The concentration of each element $i$ in the digest sample was determined based on the formula (1):

1 $$C_{i} = \frac{{C_{IS} \cdot N_{i} }}{{N_{IS} \cdot S_{i}^{r} }}$$

where:

${C}_{i}$—concentration of the element $i$ in the digest sample $\left[ {{{\mu g/g}}} \right]$, $C_{IS}$—concentration of the added internal standard (Ga) in the digest sample $\left[ {{{\mu g/g}}} \right]$, ${N}_{i}$—number of counts in fluorescence line for the element $i$ in the spectrum of the digest sample $\left[ {{\text{cts}}} \right]$, $N_{IS}$—number of counts in fluorescence line for Ga in the spectrum of the digest sample $\left[ {{\text{cts}}} \right]$, $S_{i}^{r}$—relative sensitivity for the element $i$.

The relative sensitivity coefficients of the elements, defined as the ratio of the sensitivity of a given element to the sensitivity of the internal standard, were determined based on calibration measurements of standard solutions using the formula (2):

2 $$S_{i}^{r} = \frac{{S_{i} }}{{S_{IS} }} = \frac{{\frac{{N_{i}^{s} }}{{C_{i}^{s} }}}}{{\frac{{N_{IS}^{s} }}{{C_{IS}^{s} }}}} = \frac{{C_{IS}^{s} \cdot N_{i}^{s} }}{{N_{IS}^{s} \cdot C_{i}^{s} }}$$

where:

$S_{i}^{r}$—relative sensitivity for the element $i$, $S_{i}$—sensitivity for the element $i$, $S_{IS}$—sensitivity for the internal standard (Ga), $C_{i}^{s}$—known concentration of the element $i$ in the standard solution $\left[ {{{\mu g/g}}} \right]$, $C_{IS}^{s}$– concentration of Ga in the standard solution $\left[ {{{\mu g/g}}} \right]$, $N_{i}^{s}$—number of counts in fluorescence line for the element $i$ in the spectrum of the standard solution, $N_{IS}^{s}$—number of counts in fluorescence line for Ga in the spectrum of the standard solution.

The element concentration calculated from Eq. (1) corresponds to the volume obtained after IS addition equaled to 1.55 ml (1.5 ml of the tissue digest and 50 μl of Ga solution). To determine element concentration in the tissue digest, it is necessary to take into account the mentioned dilution as follows (3):

3 $$C_{i}^{d} = \frac{1.55}{{1.5}} \cdot C_{i}$$

where:

$C_{i}^{d}$—element concentration in the tissue digest $\left[ {{{\mu g/g}}} \right]$, *C*_*i*_—element concentration in sample diluted to 1.55 ml by addition of 50 μl of IS to 1.5 ml of the tissue digest $\left[ {{{\mu g/g}}} \right]$, $\frac{1.55}{{1.5}}$—dilution coefficient.

In turn, the element concentration in the wet mass of the organ was calculated from the Eq. (4):

4 $$C_{i}^{o} = C_{i}^{d} \cdot k$$

where:

$C_{i}^{o}$—element concentration in the wet mass of the organ $\left[ {{{\mu g/g}}} \right]$, $C_{i}^{d}$—element concentration in the tissue digest $\left[ {{{\mu g/g}}} \right]$, $k$—conversion factor $\left[ {{\text{a}}{\text{.u}}{.}} \right]$.

The conversion factor *k f*or each organ sample was calculated according to the formula (5):

5 $$k = \frac{{m_{o} + m_{a} }}{{m_{o} }}$$

where:

$m_{o}$—weight of the organ $o$ $\left[ {\text{g}} \right]$, $m_{a}$—weight of the nitric acid used for the organ $o$ digestion process $\left[ {\text{g}} \right]$.

Before quantitative comparisons of elemental data, the detection limits (LOD) of elements under interest were determined for examined organs. First, $LOD_{i}$ values were calculated according to the formula (6) for each of 216 individual TXRF spectra (24 rats × 3 organs × 3 replicates) recorded during the experiment:

6 $$LOD_{i} = \frac{{3 \cdot C_{i} \cdot \sqrt {N_{BG} } }}{{N_{i} }}$$

where:

$LOD_{i}$—detection limit of element $i$ obtained for individual TXRF spectrum $\left[ {{{\mu g/g}}} \right]$, $C_{i}$—concentration of the element $i$ in the digest sample determined with TXRF $\left[ {{{\mu g/g}}} \right]$, $N_{i}$—net peak area of the $K_{\alpha }$ line of the element $i$ for the spectrum of the digest sample $\left[ {{\text{cts}}} \right]$, $N_{BG}$—area of the background under the $K_{\alpha }$ line of the element $i$ for the spectrum of the digest sample $\left[ {{\text{cts}}} \right]$.

Then, the $LOD_{i}$ values from the spectra measured for the samples of particular organs were averaged. The calculated means and standard deviations were presented in the Table S1 of Appendix.

### Statistical analysis

To verify statistical significance of the differences in elemental concentrations observed between ketogenic and standard laboratory diet fed rat organs, Mann–Whitney *U* test was utilized. The choice of this non-parametric alternative of Student's *t* test was a result of the low size of examined animal groups that did not allow for verification of the normality of the distributions of the data. The elemental differences were examined at the significance level of 0.05 and Statistica 13.3 software (TIBCO Software Inc.) was used for statistical evaluations.

### Supplementary Information

Supplementary Information.

## Data Availability

The datasets used and/or analysed during the current study are available from the corresponding author on reasonable request.

## References

[1] Wheless JW (2008). History of the ketogenic diet. Epilepsia.

[2] Hartman AL, Gasior M, Vining EP, Rogawski MA (2007). The neuropharmacology of the ketogenic diet. Pediatr. Neurol..

[3] Nordli DR, Kuroda MM, Carroll J, Koenigsberger DY, Hirsch LJ, Bruner HJ, Seidel WT, De Vivo DC (2001). Experience with the ketogenic diet in infants. Pediatrics.

[4] Mady MA, Kossoff EH, McGregor AL, Wheless JW, Pyzik PL, Freeman JM (2003). The ketogenic diet: Adolescents can do it, too. Epilepsia.

[5] Coppola G (2002). The ketogenic diet in children, adolescents and young adults with refractory epilepsy: An Italian multicentric experience. Epilepsy Res..

[6] Bough KJ, Rho JM (2007). Anticonvulsant mechanisms of the ketogenic diet. Epilepsia.

[7] Seyfried TN, Marsh J, Shelton LM, Huysentruyt LC, Mukherjee P (2012). Is the restricted ketogenic diet a viable alternative to the standard of care for managing malignant brain cancer?. Epilepsy Res..

[8] Poff A, Koutnik AP, Egan KM, Sahebjam S, D'Agostino D, Kumar NB (2019). Targeting the Warburg effect for cancer treatment: Ketogenic diets for management of glioma. Semin. Cancer Biol..

[9] Abdelwahab MG, Fenton KE, Preul MC, Rho JM, Lynch A, Stafford P, Scheck AC (2012). The ketogenic diet is an effective adjuvant to radiation therapy for the treatment of malignant glioma. PLoS One.

[10] Nebeling LC, Miraldi F, Shurin SB, Lerner E (1995). Effects of a ketogenic diet on tumor metabolism and nutritional status in pediatric oncology patients: Two case reports. J. Am. Coll. Nutr..

[11] Ruiz Herrero J, Cañedo Villarroya E, García Peñas JJ, García Alcolea B, Gómez Fernández B, Puerta Macfarland LA, Pedrón Giner C (2020). Safety and effectiveness of the prolonged treatment of children with a ketogenic diet. Nutrients.

[12] Alves-Bezerra M, Cohen DE (2017). Triglyceride metabolism in the liver. Compr. Physiol..

[13] Sampath A, Kossoff EH, Furth SL, Pyzik PL, Vining EP (2007). Kidney stones and the ketogenic diet: Risk factors and prevention. J. Child Neurol..

[14] Tokatli MR, Sisti LG, Marziali E, Nachira L, Rossi MF, Amantea C, Moscato U, Malorni W (2022). Hormones and sex-specific medicine in human physiopathology. Biomolecules.

[15] Varlamov O, Bethea CL, Roberts CT (2015). Sex-specific differences in lipid and glucose metabolism. Front. Endocrinol. Lausanne.

[16] Klockenkämper R, von Bohlen A (2015). Total-Reflection X-Ray Fluorescence Analysis and Related Methods.

[17] Laffel L (1999). Ketone bodies: A review of physiology, pathophysiology and application of monitoring to diabetes. Diabetes Metab. Res. Rev..

[18] Grandl G, Straub L, Rudigier C, Arnold M, Wueest S, Konrad D, Wolfrum C (2018). Short-term feeding of a ketogenic diet induces more severe hepatic insulin resistance than an obesogenic high-fat diet. J. Physiol..

[19] Sindhughosa DA, Wibawa IDN, Mariadi IK, Somayana G (2022). Additional treatment of vitamin D for improvement of insulin resistance in non-alcoholic fatty liver disease patients: A systematic review and meta-analysis. Sci. Rep..

[20] Westman EC, Mavropoulos J, Yancy WS, Volek JS (2003). A review of low-carbohydrate ketogenic diets. Curr. Atheroscler. Rep..

[21] Reason SL, Godfrey RJ (2020). The potential of a ketogenic diet to minimize effects of the metabolic fault in glycogen storage disease V and VII. Curr. Opin. Endocrinol. Diabetes. Obes..

[22] Hue L, Bontemps F, Hers H (1975). The effects of glucose and of potassium ions on the interconversion of the two forms of glycogen phosphorylase and of glycogen synthetase in isolated rat liver preparations. Biochem. J..

[23] Huang TY, Goldsmith FR, Fuller SE, Simon J, Batdorf HM, Scott MC, Essajee NM, Brown JM, Burk DH, Morrison CD, Burke SJ, Collier JJ, Noland RC (2020). Response of liver metabolic pathways to ketogenic diet and exercise are not additive. Med. Sci. Sports Exerc..

[24] Frommelt L, Bielohuby M, Stoehr BJ, Menhofer D, Bidlingmaier M, Kienzle E (2014). Effects of low-carbohydrate, high-fat diets on apparent digestibility of minerals and trace elements in rats. Nutrition.

[25] Bergqvist AG (2012). Long-term monitoring of the ketogenic diet: Do's and don'ts. Epilepsy Res..

[26] Acharya P, Acharya C, Thongprayoon C, Hansrivijit P, Kanduri SR, Kovvuru K, Medaura J, Vaitla P, Garcia Anton DF, Mekraksakit P, Pattharanitima P, Bathini T, Cheungpasitporn W (2021). Incidence and characteristics of kidney stones in patients on ketogenic diet: A systematic review and meta-analysis. Diseases.

[27] Khan SR, Pearle MS, Robertson WG, Gambaro G, Canales BK, Doizi S, Traxer O, Tiselius HG (2016). Kidney stones. Nat. Rev. Dis. Primers.

[28] Kubala-Kukuś A, Arabski M, Stabrawa I, Banaś D, Różański W, Lipiński M, Majewska U, Wudarczyk-Moćko J, Braziewicz J, Pajek M, Góźdź S (2017). Application of TXRF and XRPD techniques for analysis of elemental and chemical composition of human kidney stones. X-Ray Spectrom..

[29] Massey LK, Roman-Smith H, Sutton RA (1993). Effect of dietary oxalate and calcium on urinary oxalate and risk of formation of calcium oxalate kidney stones. J. Am. Diet. Assoc..

[30] Prasad AS, Bao B (2019). Molecular mechanisms of zinc as a pro-antioxidant mediator: Clinical therapeutic implications. Antioxid. Basel.

[31] Mondola P, Damiano S, Sasso A, Santillo M (2016). The Cu, Zn superoxide dismutase: Not only a dismutase enzyme. Front. Physiol..

[32] Arsyad A, Idris I, Rasyid AA, Usman RA, Faradillah KR, Latif WOU, Lubis ZI, Aminuddin A, Yustisia I, Djabir YY (2020). Long-term ketogenic diet induces metabolic acidosis, anemia, and oxidative stress in healthy wistar rats. J. Nutr. Metab..

[33] Watanabe M, Tuccinardi D, Ernesti I, Basciani S, Mariani S, Genco A, Manfrini S, Lubrano C, Gnessi L (2020). Scientific evidence underlying contraindications to the ketogenic diet: An update. Obes. Rev..

[34] Martines AM, Masereeuw R, Tjalsma H, Hoenderop JG, Wetzels JF, Swinkels DW (2013). Iron metabolism in the pathogenesis of iron-induced kidney injury. Nat. Rev. Nephrol..

[35] Nazarewicz RR, Ziolkowski W, Vaccaro PS, Ghafourifar P (2007). Effect of short-term ketogenic diet on redox status of human blood. Rejuven. Res..

[36] Ganz T, Nemeth E (1823). Hepcidin and iron homeostasis. Biochim. Biophys. Acta.

[37] Ganz T (2003). Hepcidin, a key regulator of iron metabolism and mediator of anemia of inflammation. Blood.

[38] Greco T, Glenn TC, Hovda DA, Prins ML (2016). Ketogenic diet decreases oxidative stress and improves mitochondrial respiratory complex activity. J. Cereb. Blood Flow Metab..

[39] Parry HA, Kephart WC, Mumford PW, Romero MA, Mobley CB, Zhang Y, Roberts MD, Kavazis AN (2018). Ketogenic diet increases mitochondria volume in the liver and skeletal muscle without altering oxidative stress markers in rats. Heliyon.

[40] Jain SK, McVie R, Bocchini JA (2006). Hyperketonemia (ketosis), oxidative stress and type 1 diabetes. Pathophysiol..

[41] Ishii T, Sugita Y, Bannai S (1987). Regulation of glutathione levels in mouse spleen lymphocytes by transport of cysteine. J. Cell. Physiol..

[42] Rotruck JT, Pope AL, Ganther HE, Swanson AB, Hafeman DG, Hoekstra WG (1973). Selenium: Biochemical role as a component of glutathione peroxidase. Science.

