# Supplementary material for: Ketogenic diet influence on the elemental homeostasis of internal organs is gender dependent
Source: Sci Rep. 2023 Oct 27;13:18448. doi: 10.1038/s41598-023-45611-4 (PMC10611712; doi:10.1038/s41598-023-45611-4)
Supplement: Supplementary file 1 — Supplementary Information. [file 41598_2023_45611_MOESM1_ESM.docx]

Appendix

Table S1. The averaged values of detection limits (LOD) together with their standard deviations [μg/g] for measured elements and examined organs.

|  |  | **P** | **S** | **K** | **Ca** | **Fe** | **Cu** | **Zn** | **Se** |
| --- | --- | --- | --- | --- | --- | --- | --- | --- | --- |
| **Liver** | **LOD** | 12.87 | 5.50 | 1.774 | 0.344 | 0.120 | 0.0628 | 0.0579 | 0.0373 |
|  | **SD** | 0.77 | 0.35 | 0.094 | 0.029 | 0.011 | 0.0044 | 0.0040 | 0.0027 |
| **Kidney** | **LOD** | 16.11 | 6.86 | 2.15 | 0.378 | 0.1051 | 0.0670 | 0.0604 | 0.0376 |
|  | **SD** | 0.62 | 0.26 | 0.11 | 0.017 | 0.0043 | 0.0025 | 0.0022 | 0.0015 |
| **Spleen** | **LOD** | 20.15 | 8.54 | 3.03 | 0.541 | 0.180 | 0.0907 | 0.0835 | 0.0525 |
|  | **SD** | 0.94 | 0.36 | 0.14 | 0.033 | 0.016 | 0.0061 | 0.0048 | 0.0049 |


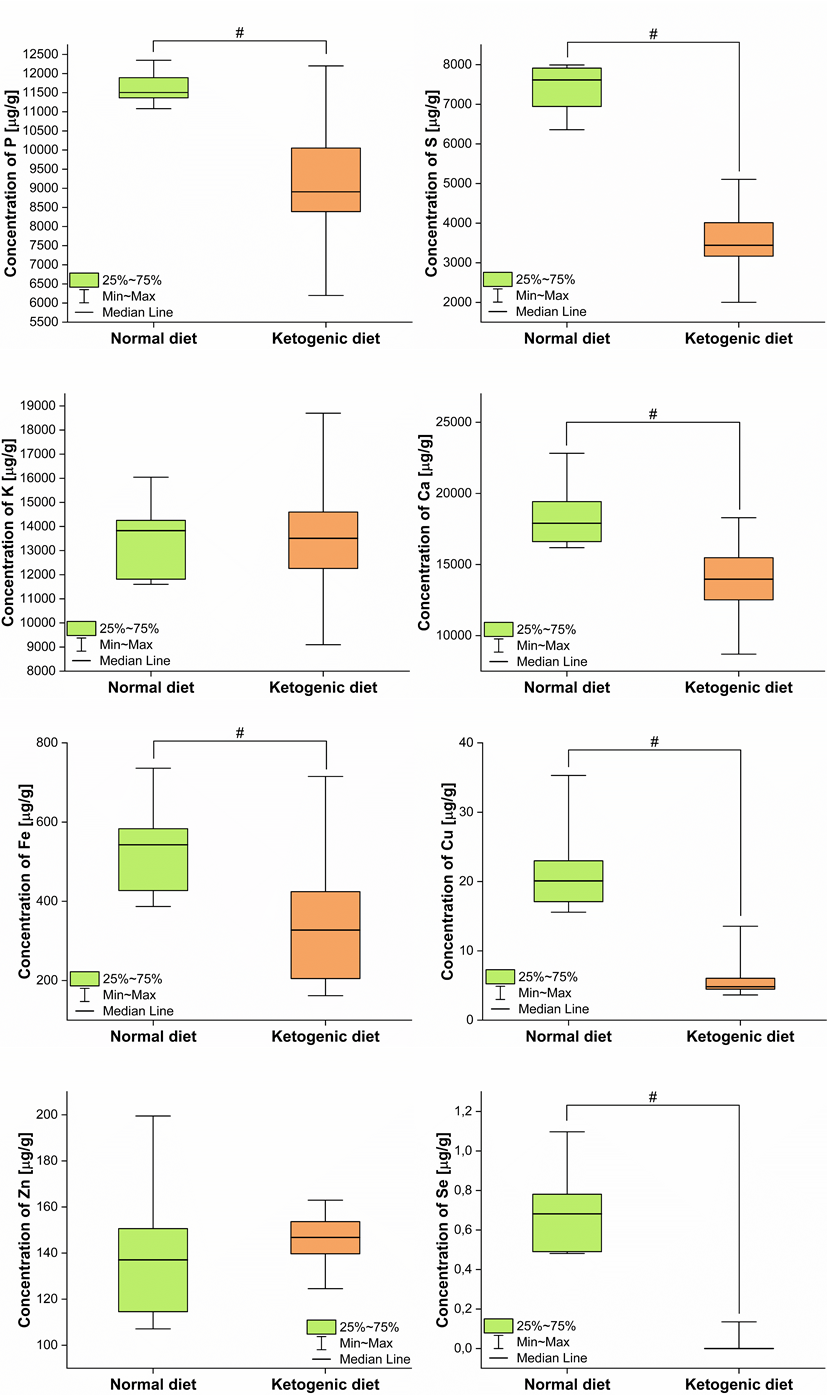


Figure S1. The comparison of the elemental composition of ketogenic and standard diet. Box-and-whiskers plots present the ranges of element concentrations [μg/g] obtained based on the TXRF measurements of 6 samples (200 mg) taken from initially homogenized fodder. Median, interquartile range and minimal-maximal values are marked as a line, a box and whiskers, respectively. The statistically significant differences determined with Mann-Whitney *U* test (*p*-value<0.05) between ketogenic and normal fodder are signed with #.
